# Remembering the Pain of Childhood: Development, Impact, and Intervention

**DOI:** 10.1080/24740527.2017.1329272

**Published:** 2017-05-22

**Authors:** Melanie Noel

**Affiliations:** Department of Psychology, University of Calgary and Alberta Children’s Hospital Research Institute, Calgary, Alberta, Canada

Pain is an inevitable part of in childhood. If poorly managed, acute pain can have adverse effects on children’s development and lead to fears and avoidance of medical care. Chronic pain in childhood is very prevalent (affecting 15-40% of youth), costly (11.8 billion USD/year), and often persists into adulthood and heightens risk for mental health disorders. Therefore, it is important to understand modifiable factors, such as pain memories, that underlie trajectories of pediatric pain. Dr. Noel’s research has documented the powerful role of memories of pain in children’s pain experiences. Children who have high fears and anxiety tend to develop distressing memories of pain, which place them at higher risk for developing pain problems in the future. Importantly, pain-related fears and memories can be modified and reframed through relatively simple language based interventions to improve pain outcomes and therefore hold promise for preventing pain problems from developing in the first place. Dr. Noel will present her program of research examining the roles of children’s memories in their experiences of acute (needle procedures) and chronic pain, as well as in the transition of pain from an acute to chronic state in youth undergoing surgeries. Solidly rooted in developmental models of pain memories and pain that she co-developed, Dr. Noel will describe how she uses innovative lab-based and clinical methods in her research with preschoolers to adolescents to examine how fears and children’s attention and memory biases influence the co-occurrence of fears and pain over time.

